# Past and Future of Phage Therapy and Phage-Derived Proteins in Patients with Bone and Joint Infection

**DOI:** 10.3390/v13122414

**Published:** 2021-12-02

**Authors:** Tristan Ferry, Camille Kolenda, Thomas Briot, Aubin Souche, Sébastien Lustig, Jérôme Josse, Cécile Batailler, Fabrice Pirot, Mathieu Medina, Gilles Leboucher, Frédéric Laurent, on behalf of the Lyon BJI Study Group, on behalf of the PHAGEinLYON Study Group

**Affiliations:** 1 Hospices Civils de Lyon, 69004 Lyon, France; camille.kolenda@chu-lyon.fr (C.K.); thomas.briot@chu-lyon.fr (T.B.); aubin.souche@chu-lyon.fr (A.S.); sebastien.lustig@gmail.com (S.L.); jerome.josse@univ-lyon1.fr (J.J.); cecile.batailler@chu-lyon.fr (C.B.); fabrice.pirot@univ-lyon1.fr (F.P.); mathieu.medina@chu-lyon.fr (M.M.); gilles.leboucher@chu-lyon.fr (G.L.); frederic.laurent@univ-lyon1.fr (F.L.); 2 Université Claude Bernard Lyon 1, 69100 Villeurbanne, France; 3 Centre de Références des IOA Complexes de Lyon, CRIOAc Lyon, 69004 Lyon, France; 4 StaPath Team, Centre International de Recherche en Infectiologie, CIRI, Inserm U1111, CNRS UMR5308, ENS de Lyon, UCBL1, 69008 Lyon, France; 5 Laboratoire de Recherche et Développement de Pharmacie Galénique Industrielle, Faculté de Pharmacie, EA 4169 “Fonctions Physiologiques et Pathologiques de la Barrière Cutanée”, Université Claude-Bernard Lyon 1, 8, Avenue Rockefeller, CEDEX 08, 69373 Lyon, France

**Keywords:** bacteriophages, phage therapy, bone and joint infection, osteoarticular infection, prosthetic joint infection, osteomyelitis, lysin, phage-derived enzyme, ANSM drug-safety agency, compassionate use

## Abstract

Phage-derived therapies comprise phage therapy and the use of phage-derived proteins as anti-bacterial therapy. Bacteriophages are natural viruses that target specific bacteria. They were proposed to be used to treat bacterial infections in the 1920s, before the discovery and widespread over-commercialized use of antibiotics. Phage therapy was totally abandoned in Western countries, whereas it is still used in Poland, Georgia and Russia. We review here the history of phage therapy by focusing on bone and joint infection, and on the development of phage therapy in France in this indication. We discuss the rationale of its use in bacterial infection and show the feasibility of phage therapy in the 2020s, based on several patients with complex bone and joint infection who recently received phages as compassionate therapy. Although the status of phage therapy remains to be clarified by health care authorities, obtaining pharmaceutical-grade therapeutic phages (i.e., following good manufacturing practice guidelines or being “GMP-like”) targeting bacterial species of concern is essential. Moreover, multidisciplinary clinical expertise has to determine what could be the relevant indications to perform clinical trials. Finally “phage therapy 2.0” has to integrate the following steps: (i) follow the status of phage therapy, that is not settled and defined; (ii) develop in each country a close relationship with the national health care authority; (iii) develop industrial–academic partnerships; (iv) create academic reference centers; (v) identify relevant clinical indications; (vi) use GMP/GMP-like phages with guaranteed quality bioproduction; (vii) start as salvage therapy; (vii) combine with antibiotics and adequate surgery; and (viii) perform clinical trials, to finally (ix) demonstrate in which clinical settings phage therapy provides benefit. Phage-derived proteins such as peptidoglycan hydrolases, polysaccharide depolymerases or lysins are enzymes that also have anti-biofilm activity. In contrast to phages, their development has to follow the classical process of medicinal products. Phage therapy and phage-derived products also have a huge potential to treat biofilm-associated bacterial diseases, and this is of crucial importance in the worldwide spread of antimicrobial resistance.

## 1. Background

### 1.1. Bacteriophage Life Circle

**Figure 1:**
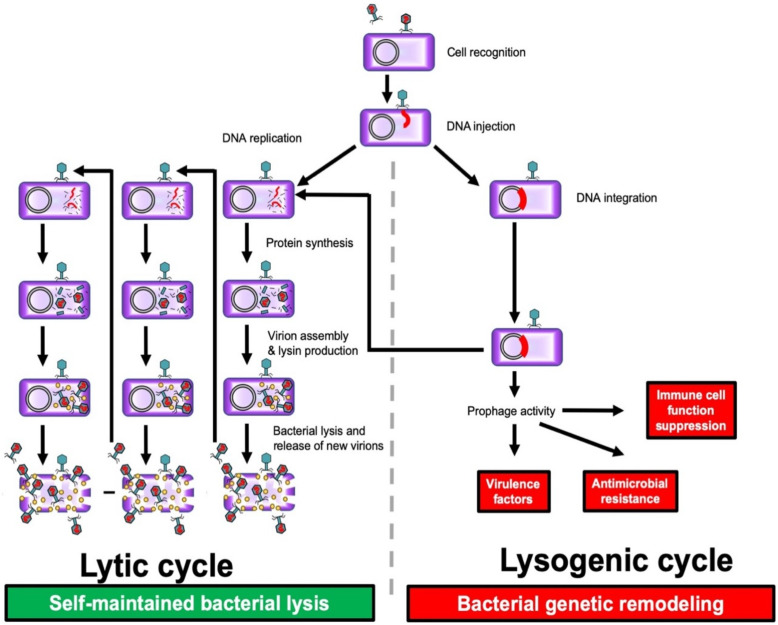
The two phage cycles: lysogenic, not causing bacterial lysis but inducing genetic remodeling and possible acquisition of genes heightening pathogenicity; and lytic, leading to self-maintained bacterial lysis, with production of lysin that disrupts the bacterial cell wall and facilitates phage dissemination; Purple: Bacteria; Green: Bacteriophage; Red: Phage DNA; Yellow: Lysin.

Bacteriophages (phages for short) are viruses that target bacteria [1,2]. Phage diversity is remarkable at the nucleotide sequence level, but more than 95% of described species belong to the *Myoviridae*, *Podoviridae* and *Siphoviridae* families, with conservative structural proteins that form viral particles [3]. Phages are uniquely interconnected with the evolution of bacterial life on earth and have undergone multiple events of genetic exchange in response to selective pressures, which also drives their diversity. There has been phage-bacteria co-evolution since the appearance of prokaryotes on earth. Phages are highly prevalent in the environment, especially in aqueous media such as salt or fresh water, drains and soil [1,2,4]. They play a major role in bacteriological ecology in nature. Each bacteriophage is mostly specific to bacterial species or even specific to a strain, which consequently means as a therapeutic agent they have no deleterious effect on microbiomes, a fact that is in huge contrast with the use of antibiotics, even narrow spectrum molecules [5]. Two different phage lifestyle modes have been distinguished: the lysogenic cycle, and the lytic cycle, corresponding to the action of lysogenic phages or of lytic phages. First of all, each type of phage adheres to the bacterium and injects its genome. During the lysogenic cycle, the genome of lysogenic phages can be integrated in the bacterial genome (prophages), thus promoting genetic exchanges between bacteria, including genes encoding toxins, or genes facilitating the escape of the host’s immunity. The integrated phage DNA can then be excised from the bacterial genome under conditions of stress, to infect another bacterium belonging to the same species, giving it these potential advantages. During the lytic cycle, a lysogenic phage could become lytic, or some other phages may have exclusively lytic activity. Lytic phages hijack the cell machinery to produce hundreds of new virions, with lysis of the bacterial host by production of different proteins such as a lysin (also called endolysin) that has enzymatic activity and disrupts the bacterial cell wall, allowing the release of progeny virions from the lysed cell. This can give rise to an exponential self-maintaining phenomenon, with the virions replicating until there is nothing left of the host (Figure 1) [1,2,4]. Peptidoglycan hydrolases and polysaccharide depolymerases are another group of phage-derived enzymes that facilitate phage attachment to the bacteria and the injection of phage genetic material [6].

### 1.2. Antibiotics and Phages as Two Different but Synergistic Products That Target Bacteria in the Environment, the Combination of the Two Limiting the Risk of Acquisition of Antimicrobial and Phage Resistance

In the environment, bacteria are continuously exposed to antibiotics and phages, and have ancestral abilities to develop resistance. Some antibiotics are naturally produced in nature, such as penicillin by Penicillium molds (mainly *P. chrysogenum* and *P. rubens*), streptomycin by *Streptomyces griseus*, and vancomycin by *Amycolatopsis orientalis* [7]. Since the purification of penicillin in the 1940s and the ability in recent decades to produce large amounts of synthetic antibiotics, antimicrobial resistance (by horizontal transfer or de novo mutations) has widely emerged and is now a threat to humanity. Various mechanisms of antimicrobial resistance have been described, such as the production of enzymes that destroy antibiotics, the modification of the target that is no longer recognized by the antibiotic, and the efflux pump that promotes the ejection of the antibiotic from the bacterium [7,8]. Phages are abundant in nature, and bacteria have developed several mechanisms to become resistant to lytic phages, such as prevention of phage entry, intracellular destruction of the phage genome, abortive phage infection and interference with phage assembly [9]. To date, several data suggest that antibiotics and phages have synergistic activity: i.e., their total effect is much greater than each individual action, in terms of efficacy, but also in terms of prevention of acquisition of antimicrobial resistance, and phage resistance [10,11,12]. Even if this phage–antibiotic synergy seems not to be universal and may be driven by a unique combination of antibacterial mechanism of action and stoichiometry of phages and antibiotics, there is a rationale for phage–antibiotic combination in difficult-to-treat bacterial infections, such as bone and joint infections [10,11,12,13].

## 2. Lessons Learned from the History of Phage Therapy

### 2.1. F. d’Hérelle

**Figure 2:**
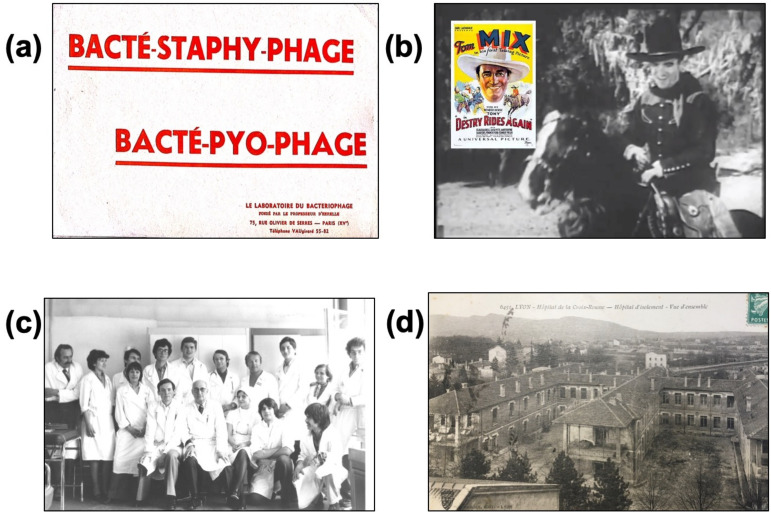
In the 1930–1940s, “ready-made” phage cocktails dedicated to specific clinical syndromes were produced and marketed by the Bacteriophage Laboratory (**a**). As these “ready-made” cocktails were considered not fully and constantly active, personalized phage therapy based on demonstrating the activity of the phage on the patient’s strain was developed to treat the American Western actor Tom Mix, who was cured during the movie *Destry Rides Again* (1932) that came out 1 year after he received abdominal phage therapy injection for life-threatening peritonitis (**b**). In the 1970s, Pr. Bertoye’s team (**c**) at the Infectious Diseases Clinic of the Lyon Croix-Rousse Hospital (**d**) identified patients in clinical failure for treatment, in partnership with the Lyon Institut Pasteur. Potentially active phages were selected and trained before use. This “personalized medicine” was used to treat 70 patients a year. Original pictures c and d are the property of T. Ferry, and have already been published in the journal *Virologie* (John Libbey Eurotext).

Phage therapy consists in using strictly lytic phages to treat bacterial infections. The concept was first described by the French microbiologist Félix d’Hérelle (1873–1949). Working in the Institut Pasteur in Paris in 1917, he showed that phages with specific activity against Shigella were found in the stool of patients who recovered from dysentery implicating Shigella. He isolated these phages and prepared a solution able to cure shigellosis by oral intake. Phage therapies were later developed against pathogens responsible for digestive and skin infections, such as streptococci and staphylococci. The phages were produced in the private Bacteriophage laboratory founded by d’Hérelle in France, and in the Eliava Institute in Tbilisi (Georgia), which he had created with his student Georges Eliava. Phages were administered orally for digestive infections and topically for skin infections. Cocktails (i.e., mixes of several bacteriophages) designed to treat different clinical syndromes were developed, such as Bacté-intesti-phage for infantile colitis and diarrhea, and Bacté-pyo-phage for suppurative skin infections such as felon, phlegmon and infected wounds (Figure 2a). F. d’Hérelle was the inventor of “ready-made” (“prêt-à-porter”) cocktails, containing different phages targeting relevant bacterial species according to the epidemiological context, but phage stability within the cocktail was unsure. At the end of 1920s, d’Hérelle was recruited by Yale University, to develop phage therapy in the USA [2]. In 1931, in the annual report of the New York Academy of Medicine, he published an article titled “Bacteriophage as a treatment in acute medical and surgical infections” [14]. He reported his experience in the treatment of staphylococcal infections, mostly furonculosis and phlegmons, with direct injection of phages at the infection site, and also in patients with osteomyelitis.

### 2.2. R.N. Smith and E.W. Schultz

In the 1930–1940s in the USA, two different events clearly divided the medical community about the efficacy of phage therapy. First of all, phages were considered as ineffective medication, as reported by several papers in the *The Journal of the American Medical Association*, even if several pharmaceutical companies such as Eli Lilly, E. R. Squibb and Abbott began to produce and widely sell “ready-made” phages. In fact, the quality of the medication and the stability of the phages were particularly poor, so much so that no phage could be detected in the preparations: “Phages obtained from commercial pharmaceutical houses have proved to be inert by the time they reach their destination” [15,16,17]. The opposite was found in the medical treatment of Tom Mix, who was a famous Western actor who was critically ill due to peritonitis with an intraabdominal abscess. R.N. Smith, Mix’s physician in Los Angeles, had no antibiotics to treat his patients. He heard of a microbiologist called E.W. Schultz who personally prepared phages to treat patients with urinary tract infections [18]. Smith asked for phages from Schultz, who refused to send a “ready-made” cocktail, as for him “whole-hearted cooperation” was needed with personalization of the phage therapy, to avoid lack of efficacy. Smith sent the infecting strain to Schultz who selected active phages that were sent back and then injected by catheter directly in the abdomen of Tom Mix, whose physical condition quickly improved (Figure 2b). This was a typical “made to measure” (“*sur-mesure*”), or “personalized” phage treatment, with selection of phages active against the infecting strain, the premise of the “phagogram”. Several other patients with similar clinical conditions were treated in a same way by Smith [19].

### 2.3. A. Raiga-Clémenceau

André Raiga-Clémenceau, another student of d’Hérelle’s, reported phage therapy for bone and joint infection in the 1930s. He underlined the complexity of treating these infections and the failure of phage therapy in osteitis and chronic osteomyelitis. He went as far as to say that, “At the bone necrosis stage, it can succeed only in arresting progression, but can do nothing for the bone that death has deprived of vascularization; this bone will be sequestered and the lesion is now a matter only for surgery. To do anything else is, in my opinion, to commit an error of therapeutic indication” [20]. After World War II and the widespread use of penicillin and its derivatives, which were stable chemically and had a wide spectrum of action against most of the bacteria responsible for infections, the use of phage therapy totally collapsed, as the need to individualize a virus-based therapy was too complex in comparison with the easy use of antibiotics.

### 2.4. Lyon Pasteur Institute and the Infectious Diseases Clinic of the Lyon Croix-Rousse Hospital

In the 1950s and 1960s, several patients underwent phage therapy in Lyon, France, thanks to local expertise developed up until the 1980s by the Lyon Pasteur Institute, and Dr. Guillermet, Pr. Sédallian and Pr. Bertoye in the Infectious Diseases Clinic of the Lyon Croix-Rousse Hospital (Figure 2c,d). At this time, the Lyon Institut Pasteur was in charge of microbiology for the Lyon Hospitals Board and phage discovery in human samples and phage banking in the Institut Pasteur enabled treatment of several types of severe infection such as meningitis or burn wound infection caused by *P. aeruginosa* [21,22,23]. In articles published in the 1960s, Pr. Bertoye further reported “phage training” on the patient’s isolate ahead of administration to enhance efficacy, in a personalized approach to phage therapy, that was also mentioned by Raiga in the past [23]. The aim of phage training is to facilitate the occurrence of mutations induced by the co-evolution of the lytic phage with a bacterium, to increase the lytic activity and the replication process of the phage into the targeted bacteria. These were “personalized” phages: bank phages were assessed on a “phagogram” then “trained”, to enhance lysis and self-amplification [21,22,23]. At the peak of phage therapy in Lyon, 65 patients were treated in 1976, 33 with phages targeting *P. aeruginosa*, 19 with phages targeting an enterobacterium, and 13 with phages targeting *S. aureus* [24]. The Lyon Institut Pasteur closed in the 1980s, and all the bank phages and know-how were lost. In 1979, Lang et al. reported 7 cases of recurrent chronic bone and joint infections treated by phage therapy, including 1 *P. aeruginosa* joint implant infection, managed by surgery, local injection of preselected phages and antibiotic therapy, in the hope of maximizing success [25].

### 2.5. Eastern Experience in the Eliava Institute in Georgia, in the USSR and in the Hirszfeld Institute in Poland

Before and just after the World War II, it is important to note that phage therapy development was mainly supported by countries located in the East of Europe. The Eliava Institute in Georgia, which was founded by F. d’Herelle and G. Eliava in 1923, is still open and phage therapy is still used as traditional medicine in this country [26]. At the same time in the 1930s, phage therapy became well established in the infrastructure of Soviet microbiological institutes [27]. During World War II, bacteriophage preparations were developed in practice by physicians and military authorities, and were widely used, notably during the Battle of Stalingrad. During the Cold War, Soviet scientists protected phage therapy from contemporary Western criticism, and the bacteriophage development and research program continued in Georgia [27]. In Poland, Bronislawa Feijgin, who graduated from Paris medical school in 1914, worked also on bacteriophages and then reported her experience in this country. During the post-World War II period, Ludwick Hirszfeld developed phage therapy in Poland and founded the Hirszfeld Intitute, where a Phage Therapy Unit was created in 2005 [28].

### 2.6. Contemporary Period

#### 2.6.1. Villeneuve Saint Georges Team

**Figure 3:**
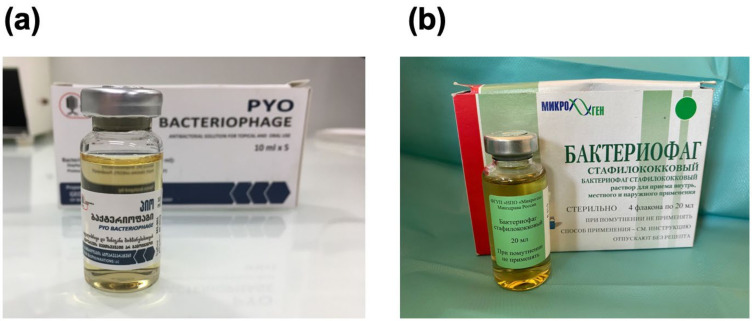
“Ready-made” cocktails currently produced in Eastern European countries: the Pyo-bacteriophage cocktail of the Eliava institute, Georgia (**a**) containing various titers of phages targeting *Staphylococcus* spp., *Streptococcus* spp., *E. coli*, *Pseudomonas aeruginosa* and Proteus spp.; and a bacteriophage targeting *Staphylococcus* spp. (**b**) produced in Russia by the company Microgen.

More recently, Patey et al., in the Villeneuve Saint Georges team, reported their experience with phage therapy in 15 French patients between 2006 and 2018, using phages produced in Russia or the Eliava Institute (“ready-made” cocktails, theoretically banned for import or use in France) (Figure 3a,b) [29]. Nine of the patients had resistant bone and joint infection, including post-traumatic osteomyelitis and implant infection. Phages were administered intraoperatively and postoperatively on exposed bone or injected into the surgical drains. Some clinical success was reported, but the bone and joint infections and the medical and surgical treatments were very heterogeneous.

#### 2.6.2. Pherecydes Pharma

In France, Pherecydes Pharma, founded in 2006, produces purified phages for use within the European Union, which entails adhering to “good manufacturing practice” (GMP) guidelines and the requirements of the French National Agency for Medicines and Health Products Safety (ANSM), unlike for Russian or Georgian phages, which may contain pyogenic substances (endotoxins or other bacterial debris) or tempered phages with integration potential or immune suppression activity [30]. Pherecydes Pharma designed and tested phages targeting *P. aeruginosa* and secured ANSM authorization for PHAGOBURN, a European therapeutic trial in burn patients [31], the first randomized therapeutic trial using GMP-standard phages. The cocktail was produced by Clean Cells (France) with a complete description of each manufacturing step and external validations that were reviewed by ANSM in an Investigational Medicinal Product Dossier. Published in 2019, this clinical trial showed reduced bacterial load in both the phage group and in the control group receiving silver sulfadiazine, the antibacterial activity of which has been underestimated. Moreover, the stability of the phage cocktail used for this trial unfortunately declined during time, explaining at least in part the lower than expected activity of the phages. Despite negative results, the trial demonstrated feasibility, optimizing and validating bioproduction of purified phages. At the same time, a partnership between Pherecydes Pharma and the Lyon Hospitals Board (Hospices Civils de Lyon) selected phages targeting *S. aureus* and active on most strains implicated in bone and joint infection, with the possibility of GMP production for use in clinical trials and/or with a temporary authorization for use. Preclinical efficacy data for the selected anti-*S. aureus* phage cocktail were obtained in vivo and in vitro: a single injection of this cocktail in an animal model of non-diabetic and diabetic foot infections was at least as effective as a single systemic injection of antibiotics in reducing bacterial burden; this cocktail was highly active against *S. aureus* embedded in biofilm, and was synergistic with vancomycin [32,33].

#### 2.6.3. Compassionate Use of Phages

Since the launch of PHAGOBURN in 2015, the ANSM has received requests for “compassionate use” of phages against *P. aeruginosa* and *S. aureus* in patients in therapeutic failure in a life-threatening infection: i.e., experiencing relapse despite well-conducted conventional antibiotic treatment. The Helsinki Declaration of the World Medical Association, reprinted in *JAMA* in 2013, states that: “In the treatment of an individual patient, where proven interventions do not exist or other known interventions have been ineffective, the physician, after seeking expert advice, with informed consent from the patient or a legally authorized representative, may use an unproven intervention if in the physician’s judgement it offers hope of saving life, re-establishing health or alleviating suffering” [34]. The patient’s physician can thus call upon the drug safety agency if use of phages is considered likely to be beneficial. In Belgium, patients are occasionally treated at the Queen Astrid Military Hospital (QAMH), which was the first Belgian hospital to reinitiate phage therapy in 2007 under the umbrella of the Declaration of Helsinki [35]. At the present time, the majority of phage therapy requests to the QAMH originated from neighboring countries such as the Netherlands, which accounts for two-thirds of the requests. In France, the health authority “accompanies” on a case-by-case basis the administration within France of phages not strictly meeting GMP standards but with comparable quality produced by Pherecydes Pharma or by the QAMH, with preparations administered under the entire responsibility of the prescribing physician and the hospital pharmacist. Feedback from a Temporary Specialized Scientific Committee (CSST) on phage therapy was arranged by the ANSM in 2019, and the conclusion was the following: “All of the issues raised by phage therapy have led to a plea for the setting up of a national platform for the orientation and validation of the use of phages in order to manage the use of phages in France and which could eventually work towards the implementation of academic production of phages for clinical use in France from a phage library. In view of the critical issues at stake, it is expected that this platform will be set up at a ministerial level with the authorities involved in the organization of care” [36]. In the 2015–2019 period, out of 45 requests to the ANSM, only 12 patients were treated, due to lack of available phages, clinical deterioration in the patient, incompatible administration route, or clinical improvement in the patient; 7 of these were treated in the Lyon referral center for the management of complex bone and joint infection (CRIOAc Lyon; http://www.crioac-lyon.fr (accessed on 22 November 2021)) [37,38,39]. Following the CSST meeting in 2019, a technical report was produced to support the secured use of phages in France [40].

#### 2.6.4. Creation of a Dedicated Program “PHAGE*in*LYON” for Phage Therapy in CRIOAc Lyon

In the CRIOAc Lyon center, a dedicated team has been set up to manage complex bone and joint infection, and the objective of the PHAGE*in*LYON program is to use this multidisciplinary approach to propose innovative treatment such as phage therapy, in the most relevant indications [1,39]. In this center to the end of 2021, 30 patients had been treated, most for life- or function-threatening recurrent complex bone and joint infection [37,38,41,42,43]. Of note, these patients were referred mainly from the Auvergne–Rhône–Alpes region (the central and eastern region of France from which Lyon is the capital), but also from all over France [43]. One model of this program is the Innovative Phage Applications and Therapeutics (IPATH) center in the USA, which has been set up in parallel.

#### 2.6.5. Creation of the San Diego (CA) Center for Innovative Phage Applications and Therapeutics (IPATH)

Recently, in the USA, the FDA’s attitude was transformed in the light of the Tom Patterson case [24]. This patient presented with a multidrug-resistant *Acinetobacter baumannii* suppurative abdominal infection. Active phages were purified and also trained for individual treatment. After FDA approval, the patient was authorized to receive an “eIND” (Emergency Investigational New Drug) for compassionate use. The 2018 report on Antimicrobial Agents and Chemotherapy showed the importance of using a ready-made active cocktail to avoid the emergence of phage-resistant strains, and of implementing phage training and iterative injection [44]. Subsequently, a dedicated phage therapy center has received official funding: The San Diego (CA) Center for Innovative Phage Applications and Therapeutics (IPATH). We believe that the IPATH model of reference center for the development of phage therapy could be replicated at national level in each country. Indeed, in order to develop phage therapy, national academic reference centers could be set up in each European country, in direct link with their respective national health authorities, with the creation of an interactive European network that will favor the sharing of experiences and the performance of multicentric clinical trials in this field [43,45].

## 3. Is Bone and Joint Infection a Relevant Indication for Phage Therapy?

Bone and joint infections have the reputation of being the most difficult bacterial infections to treat. They are often post-traumatic, or hip or knee implant infections. Various bacterial persistence mechanisms are involved, such as intracellular persistence within osteoblasts or synovial cells, or biofilm production [46], implicated in chronicity and recurrence, and usually requiring heavy surgery with implant exchange. In some patients, this is not feasible, and “conservative” procedures may be proposed, with long-course antibiotic therapy [47,48,49,50,51,52,53,54,55]. Failures, however, occur, with persistent clinical signs of infection, superinfection by other bacteria, or acquired resistance. There are good reasons for envisaging phage therapy in bone and joint infection: (i) these are the most severe nosocomial infections, frequently due to *S. aureus*, coagulase-negative staphylococci, but also *C. acnes*, streptococci spp., Enterobacteriaceae and/or *P. aeruginosa* [56]; (ii) with the emergence of antimicrobial resistance, more and more bone and joint infections are treated with less active antibiotics, or with new beta-lactam antibiotics (that have no anti-biofilm properties) such as ceftazidime-avibactam or ceftolozane-tazobactam [57]; (iii) their functional impact is devastating, and their cost to the health system no less dramatic [58,59]; (iv) in France, the CRIOAc network can validate optimal medico-surgical strategy and perform regional therapeutic trials [60]; (v) phages can act on biofilm, and in synergy with antibiotics [10,11,12,33]; and (vi) bone and joint infections are often chronic, and there is no major inconvenience in waiting a few days or weeks to identify the strain causing relapse and test its sensitivity to phages, as required by the ANSM. Prosthetic joint infection seems to be a particularly relevant indication for phage therapy, especially in patients for whom prosthesis explantation might be associated with significant loss of function. In patients without prosthesis loosening but with clinical signs of septic arthritis and positive joint aspiration, phages are easy to use as they can be injected directly into the joint cavity after debridement [38,42]. The next step is to perform clinical trials in patients with prosthetic-joint infection, to demonstrate that phages could provide added value by increasing the probability of microbiological cure and by limiting the risk of prosthesis revision.

## 4. Is Phage Therapy Feasible in the 2020s in Patients with Bone and Joint Infection?

Implementing phage therapy in a dedicated indication presupposes having firstly pharmaceutical-quality phages available, and clinical multidisciplinary expertise for their use as compassionate treatment.

### 4.1. Obtaining Pharmaceutical-Quality Phages

To obtain and to use pharmaceutical-quality phages, several steps are required. First a clear definition by the European Medicines Agency (EMA) about the status of phages is mandatory. Second, the quality controls that are mandatory before application in humans have to be determined.

#### 4.1.1. Classification

For the European Medicines Agency, phage status is not yet defined. To harmonize phage regulation in Europe, a workshop was organized on 8 June 2015 [61]. Through a collegial decision, phages were classified in the biological drugs category whereas phages do not meet all requirements of viral agents [62]. Because phages are extracted from natural sources, there may be classified in the allergen group [63]. Currently, the agency is looking for more robust evidence of bacteriophage treatments and will then discuss the scientific and regulatory status with stakeholders. Awaiting EMA’s final decision, in France, a temporary specialized scientific committee was set up by the French drug-safety agency. The committee accorded phages the status of medicines [36]. For Belgian regulations, phages are considered as active pharmaceutical ingredients (API). In fact, these discussions underline the need for clarification of quality/safety requirements and the necessity of revising the developmental path for innovative medicines, which require a more personalized approach and necessitate the incorporation of the dynamics of “phage-bacteria co-evolution” that cannot be fixed as the development of a single drug or in a fix cocktail of phages [64,65,66]. Considering evolutionary biology seems to be a crucial step for the successful development of personalized phage therapy [66].

#### 4.1.2. Quality Controls to Be Performed

As explained above, the status of phages is not well defined, and scientific data are expected about the risk/benefit ratio in various clinical indications. Phages are more and more frequently used as compassionate treatment, and future clinical trials will provide additional data about safety. To secure the use of phage therapy, for compassionate use as well as for clinical trials, some quality controls seem to be mandatory.

First of all, phages must be isolated and purified from the environment. Numerous papers describe methods for phage isolation [67,68,69,70]. Adnan et al. used water samples from different sources: rivers, springs and waste water channels in Pakistan; the exact isolation method was not specified [67]. Other papers from Belgium refer to a study by Merabishvili et al. which depicts the entire process of manufacturing a well-defined bacteriophage cocktail for use in human clinical trials [68]. They used *P. aeruginosa* strain ‘573’, isolated from bone-marrow interstitial fluid, and *S. aureus* strain ‘13 S44 S’, isolated from a burn wound, to produce phages. Different purification methods have been published and can be used by academic laboratories [67,68,70,71,72,73].

Characterization of phages for a human medical application, as carried out for chemical drugs, must include data of the raw material (or API depending on the country’s regulations). Firstly, it corresponds to phage identification (i.e., DNA sequencing, electronic microscopy, absence of lysogenic phages) and to data regarding the biopurification process (strain used for amplification, purification techniques). Finally, batch quality controls must be performed (purity, quantification, endotoxin levels, sterility, pH, absence of cellular remnants, cytotoxicity). Very few countries have specific guidelines regarding these requirements. Recently, in Belgium, since 2018, phages could be prescribed by a physician as a magistral preparation, with an official ‘definitive’ monograph under review to be included in the Belgian Pharmacopeia detailing tests and validated methods to qualify phages for human medical applications [74].

Before engaging phages in a preparation for a medical application, their stability (alone in suspension form or in cocktail) has to be evaluated. For this, various stress conditions such as temperature, light, mixing and different phage concentrations have to be challenged. If necessary, to increase the stability of the preparation, several processes could be implemented: e.g., stabilizing additives, lyophilization, microencapsulation or nanoemulsification.

Finally, after qualifying the raw material (or API), the preparation administered to the patient needs to be controlled following general pharmacopeia prescriptions (US, European, or other else) regarding parenteral preparations.

### 4.2. Multidisciplinary Clinical Expertise

**Figure 4:**
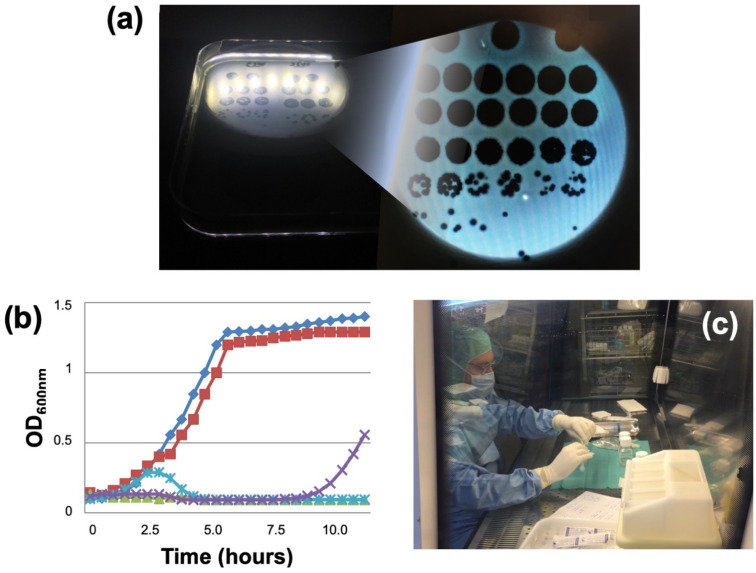
Phagogram and phage preparation techniques. PFUs visualized by spot test using solid medium and optic microscopy (**a**): For the lowest phage suspension dilutions (pure, and 10-1 to 10-3 dilutions: first lines), PFUs are confluent and uncountable. Further dilution allows some PFUs to be counted (4th and 5th lines), and the number of PFUs in the solution to be deduced. The killing assay studies temporal progression of DO600nm in liquid medium (**b**), monitoring bacterial multiplication with and without phages, indicating susceptibility/resistance: bacterial multiplication without phages (blue line with square); inactive phage (red line with square); active phage with slight delayed inhibition of the bacterial growth (turquoise line with cross); active phage, but with delayed bacterial growth (purple line with cross); fully active bacteriophage totally inhibiting bacterial growth (green line with triangle). Preparation in a Lyon hospital pharmacy of an extemporaneous phage solution in sterile conditions at time of surgery (**c**).

Phage therapy then requires a second preliminary step of multidisciplinary expertise to be sure there are no alternatives. In bone and joint infection, the expertise of CRIOAc centers is essential [60]. Other innovative treatments are available and implemented in some CRIOAcs. For example, in chronic osteomyelitis, cutting-edge medico-surgical strategies combined with devices delivering highly concentrated local antibiotics associated with systemic antibiotic therapy can be considered, especially as such devices are available on the market with CE labeling ensuring conformity to EU legislation. The lack of alternatives or their possible function- or life-threatening consequences have to be argued before the ANSM. Physicians must also do all possible to demonstrate that the clinical signs exhibited by a patient in failure are indeed due to the bacterium to be targeted—which is very complicated in chronic osteomyelitis but much easier in implant infection, where simple joint aspiration can identify the strain. Once the indication has been approved, a phagogram has to be drawn up, testing isolate susceptibility to the available phages. There are two complementary techniques, the spot test and the killing assay (Figure 4a,b), which cannot of course be routinely performed in any lab [33,37]. Phages considered “active” are sent to the hospital pharmacist in individual vials of concentrated solution, usually at 1 × 10^8^ to 1 × 10^10^ plaque-forming units (PFU)/mL. The pharmacist then prepares the phage cocktail extemporaneously under a microbiology safety cabinet (Figure 4c). The dilution depends on the endotoxin level in the purified solution. Consequently, use of highly purified phages at high concentration (1 × 10^9^ to 1 × 10^10^ PFU/mL) limits the risk of having to use phages at too low a concentration to have a biological effect. The preparation is then sent immediately to the operating room for intraoperative application. Patients also receive broad-spectrum antibiotic therapy. It is thus possible to implement phage therapy in France with purified phages, thanks to the combined efforts of the academy, industry and ANSM. These are, however, extreme clinical situations, in patients with a complex orthopedic history presenting a failure, for whom it is not possible to evaluate different administration modalities, optimal dose or administration frequency. These compassionate cases are, on the other hand, crucial to demonstrate feasibility and facilitate the optimization of the whole chain from production to administration of the phages.

## 5. What Are the Perspectives for Phage Therapy 2.0?

We can call the current trend for phage utilization “phage therapy 2.0”, which corresponds to the use of quality-controlled purified phages for compassionate use, in clinical trials, and may be used in future in validated indications. The perspectives for phage therapy 2.0 are based on the experience gained from the treatment of compassionate cases, which will help to determine the best clinical indications for phage therapy. It has to include the availability of large industrial or academic panels of pharmaceutical-quality phages, to finally perform clinical trials to demonstrate the benefit of phage therapy.

### 5.1. Compassionate Cases and Relevant Indications in Bone and Joint Infection

**Figure 5:**
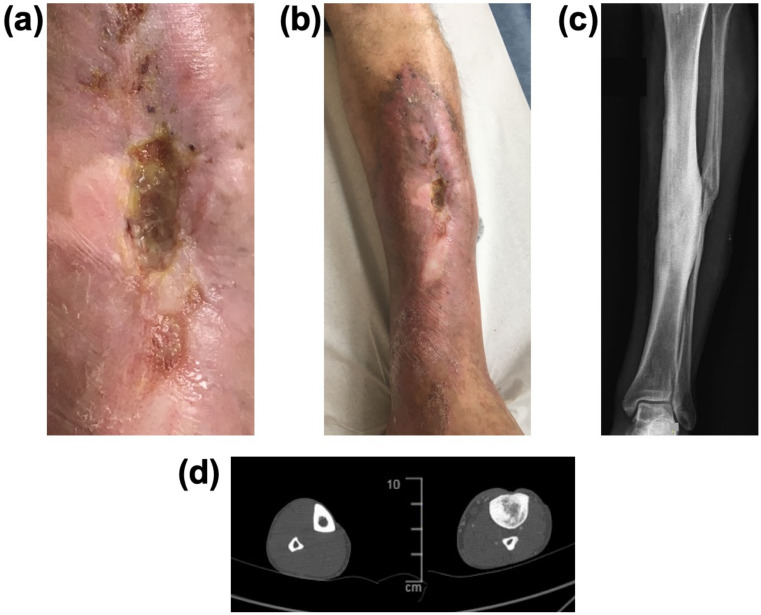
Post-traumatic left tibial chronic osteomyelitis in a French patient in failure of phage treatment administered in Georgia. A ready-made cocktail was administered locally and *per os* for several weeks. Bone exposure persisted (**a**) with large skin adherences (**b**) to underlying sclerotic bone (**c**) X-ray without any abscess or intramedullary cavity on CT-scan (**d**); transverse view of both legs; on the left the leg without infection, on the right the infected leg, with sclerotic bone and densification of the medulla). Heavy high-tech surgery in a CRIOAc reference center is preferably indicated, with bone curettage (only means of eradicating infected necrotic bone), resection of the large skin area around the exposed bone, related to chronic inflammation and unable to recover, with free-flap cover followed by prolonged antimicrobial therapy. Intraoperative and postoperative phage administration is clearly not feasible in such a patient.

Compassionate use of phage therapy using pharmaceutical-quality phages is possible in industrialized countries such as in France and Belgium, but complex [35,43,75,76]. The first cases managed in France used industrial phages targeting *P. aeruginosa* that were approved for the PHAGOBURN clinical trial [31,37]. All cases treated in France are managed in collaboration with the ANSM, and there are obstacles on the path of this treatment. The first is to properly define indications and incorporate phage therapy within the various medico-surgical strategies available. Chronic osteomyelitis is a classically difficult clinical situation, and European patients frequently end up traveling to Georgia to receive one of the Eliava Institute’s ready-made cocktails, administered locally around the infected bone and orally (considering a systemic diffusion after an oral intake) for several weeks, in the hope of slowing progression. Some patients report spectacular success without recourse to surgery or antibiotics. Others are in failure, whereas they could be cured by medico-surgical treatment in a CRIOAc (Figure 5a–d). In Belgium, a standardized multidisciplinary treatment protocol was implemented, with phage administration during surgery, and then after surgery for some days using tubes placed at the site of infection [76]. However, evaluation of such patients is quite complex as they have very heterogenous clinical situations. In our view, osteomyelitis may thus not be the best indication for phage therapy, especially for clinical trials. Prosthetic joint infection, on the other hand, is potentially a relevant indication, with the chance of leaving a functional implant in place. When infection is chronic, the implant in principle needs replacing (also called revision), which is complicated, especially in elderly patients with large prostheses, as removal is generally associated with loss of function or even death. The majority of patients managed in the Lyon CRIOAc have chronic prosthesis infection without prosthesis loosening, for whom a simple “debridement, antibiotics and implant retention” (DAIR) procedure was followed by a one-shot injection of the phage cocktail into the joint after closure [38]. This surgery is easy to perform, reproducible and particularly safe; the joint cavity may facilitate phage replication among planktonic bacteria, and the phage could target the biofilm at the prosthesis surface, facilitating the success of suppressive antimicrobial therapy. As using tubes for postoperative injection of phages incurs risk of superinfection [77], we do not plan to repeat the phage injections, but we do not know if a single shot administration is enough to start the phage multiplication process in the targeted bacteria. All these situations of early availability of phages for compassionate use have revealed the complexity of the clinical cases and highlighted the need to determine the exact indications for phage therapy among other possible strategies. Some other patients were treated in our institution with phages that target *S. aureus*; they were produced in research and development programs of the private French company Pherecydes Pharma, following the GMP-like process of purification [37,38,41,42,78]. Although these phages were not approved for clinical trials, some patients were also treated as salvage therapy, under the supervision of the French Health Ministry, based on the experience gained from the phages used in the PHAGOBURN clinical trial [31].

### 5.2. Availability of Large Industrial or Academic Panels of Purified Pharmaceutical-Grade Therapeutic Phages (i.e., Following Good Manufacturing Practice Guidelines or Being “GMP-like”)

**Figure 6:**
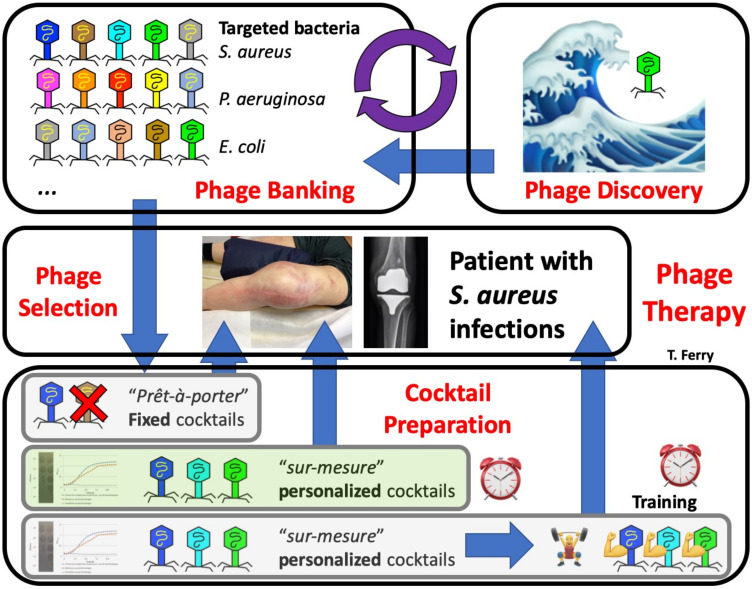
The whole process required for phage therapy: phage discovery; phage banking (that has to be implemented and maintained) with various phages targeting different bacteria; relevant clinical indication; phage therapy using a fixed cocktail, with a certain likelihood that some of the phages will not be not active; phage selection based on the phagogram (currently performed in France), with cocktail preparation and administration in hospital (green option); phage selection based on the phagogram, with phage training on the patient’s strain, which is not currently performed and is more time consuming than the previous options.

Because of the narrow spectrum of phages, generally limited to one bacterial species and often to a restricted number of strains within this species, it is important to develop collections of phages active against a large panel of clinical isolates. Phages can be isolated from various environmental sources or from clinical samples, after enrichment in liquid culture with bacterial strains (Figure 6). Their presence in these samples is then detected by applying the supernatants on the surface of agars seeded with the targeted clinical isolates. Samples triggering bacterial lysis potentially contain one or more specific phages that will be further isolated through double-layer assays. Then, the genomes of newly isolated phages have to be sequenced to identify them, and to ensure the absence of undesired genes coding for virulence or resistance genes or for integrases associated with risk of lysogenic cycle and gene transfer between bacterial strains. From these banks, phages can be selected for their activity against a clinical strain (phagogram) to optimize the composition of the cocktail which will be used to treat the patient, contrary to “ready-made” cocktails. Finally, in particular clinical situations, especially in chronic infections due to multidrug-resistant isolates also potentially resistant to phages, phage training can increase phage activity against the patient’s strains (Figure 6). In each European country, there is a clear place for academic and industrial collaboration for developing large panels of clinically utilizable phages of pharmaceutical quality. In fact, from an industrial point of view, phage therapy is limited by the possibilities of filing patents, which limits investment by pharmaceutical companies and restricts the performance of clinical trials. The development of patents is delicate first of all because phage therapy is not really an innovative treatment, as it has been described and used for a long time, before widespread use of antibiotics. However, the patent must meet certain conditions. To frame and protect the marketing of phage therapy, it would be necessary to be able to create a phage composition that had never been proposed before or to deposit precise genomic sequences characterizing each phage. The latter possibility is made difficult by the evolutive potential of phages, as they are natural products that continuously adapt to bacterial evolution. Consequently, a well characterized active phage could become obsolete and inactive with years, depending on the type of phage and on the evolutive process of the targeted bacteria. The impossibility of protecting phage treatment therefore discourages manufacturers for fear of lack of return on investment, whereas industrial purification seems to be essential for authorities to obtain pharmaceutical-quality phages. On the other hand, most academic hospitals have large collections of bacterial strains isolated from patients and are interested in developing “new” anti-infective treatments, and most have the technical ability to perform a phagogram. Developing phage discovery and phage banking requires considerable investment, but this is fundamental for the implementation of phage therapy 2.0. Creation (and funding) of a national academic phage center is considered by the French health authority to be an essential step to reach this objective.

### 5.3. Performance of Clinical Trials

Phase II/III therapeutic trials are essential, ultimately, to demonstrate the efficacy of phage therapy in each relevant clinical situation. The French CRIOAc network and its scientific committee should enable such trials to be conducted [60]. Other types of severe bacterial infection, such as intensive care infections (sepsis, pneumopathy induced by mechanical ventilation) or infectious endocarditis, are also possible indications. Application of phages by aerosol or intravenous route, associated with antibiotic therapy, could improve prognosis, but the current situation of exclusively compassionate use involves unavoidable delay: transmitting the phage strain, performing the phagogram, validating the indication, etc. Implementing phage therapy in less severe community infections such as urinary infection or in decolonization of digestive carriage of multiresistant bacteria should be considered in the long term, to combat antibiotic resistance. However, widespread use of ready-made cocktails in such indications is a far cry from the present strictly “compassionate” use. The various stakes raised by phage therapy led the ANSM to advocate setting up a national platform for guidance and validation of the use of phages [36]. Academic involvement in phage discovery, phage banking, phage training and preclinical models to shed light on anti-infectious action, phage-resistance risk, synergic action on biofilm and phage–antibiotic synergy are key elements complementing the involvement of industry. Finally, depending on what indications are judged suitable and on the preclinical models, determining optimal administration routes (intravenous, intra-articular, intravesical, oral) and dosages (once or several times daily, administration duration, treatment duration, etc.) is essential in order for this therapy to have its recognized place in the anti-infectious armamentarium [79].

## 6. What Are Phage-Derived Proteins?

Several phage-derived proteins have been identified. Peptidoglycan hydrolases and polysaccharide depolymerases are a group of phage-derived enzymes that facilitate the attachment of the phage to the bacterium and the injection of the phage genetic material [6]. They have potential in vitro lytic and antibiofilm activity, but up to now no clinical development seems to be ongoing [80]. Lysins are hydrolytic enzymes produced during the final stage of the lytic cycle by phages in order to cleave the bacterial cell wall (Figure 1) [81,82]. They are highly evolved enzymes that target the peptidoglycan, the cell wall substrate. Lysins are usually monomeric proteins that have two domains: a C-terminal domain that binds peptidoglycan, and an N-terminal domain with hydrolytic activity. As the peptidoglycan is essential for containing the internal structure of the bacterium, a hole in the peptidoglycan irreversibly causes immediate hypotonic lysis. The huge lytic activity of endolysins raised the question of purifying and using them as isolated therapeutic agents, especially against Gram-positive bacteria, as the peptidoglycan of these bacteria is not covered by an outer membrane. Phage lysins are generally species- or subspecies-specific, with a broader spectrum of action than phages. For instance, phages targeting *S. aureus* are usually not active against coagulase-negative staphylococci, whereas the lysin purified from these phages is [83]. Some companies are developing recombinant lysins, and CF-301 (Exebacase) is the most advanced staphylococcal lysin in development. The production process and the development of lysins follows the classical approval pathway of all drugs. CF-301 has a synergistic antimicrobial activity and recently a phase 2 study showed improvement in patients receiving CF-301 (one intravenous injection) in addition to standard-of-care antibiotics compared to standard-of-care antibiotics alone [81,84]. Equivalent findings have been made with lysin SAL-200 in a preclinical model, and a clinical trial in patients with *S. aureus* bacteremia is under way [85]. In the field of bone and joint infection, CF-301 demonstrated a broad spectrum of action against staphylococci, and was shown to be highly effective in clearing biofilms in different models [82]. One-shot addition of CF-301 to daptomycin in an animal model of osteomyelitis significantly reduced the bacterial load [86]. Several proposals could emerge concerning the positioning of lysins in comparison to phage therapy. One way would be to use lysins in patients with prosthetic joint infection due to coagulase-negative staphylococci, for whom explantation is not feasible: no phages active against coagulase-negative staphylococci are available, whereas CF-301 seems to have a wide activity against staphylococci. In this context, our group recently treated some patients as salvage therapy with CF-301, and we concluded that it could be used in such patients, to improve the efficacy of suppressive antibiotics and to prevent major loss of function [87]. Under the support of health authorities, clinical trials evaluating the pharmacokinetics and the safety of this approach, and then its efficacy, could be conducted in the near future.

## 7. Conclusions

The story of phage therapy is fascinating, and phage therapy has a huge potential in the field of bone and joint infection. Prosthetic joint infection seems to be a particularly relevant indication for phage therapy, especially in patients for whom prosthesis explantation might be associated with significant loss of function. Globally, “phage therapy 2.0” in Europe has to integrate the following steps: (i) follow the future status of phages, which is currently under consideration; (ii) develop in each country a close relationship with the national healthcare authority; (iii) develop industrial-academic partnerships; (iv) create academic reference centers; (v) identify relevant clinical indications; (vi) use pharmaceutical-quality phages with guaranteed quality bioproduction; (vii) start as salvage therapy; (vii) combine with antibiotics and adequate surgery; and (viii) perform clinical trials, to finally (ix) demonstrate in which clinical settings phage therapy provides benefit. For that purpose, national and international structuring initiatives are required to complete the panorama.

In France, in the CRIOAc Lyon center, a dedicated team has been set up to manage complex bone and joint infection, and the objective of the PHAGE*in*LYON program is to use this multidisciplinary approach to propose phage therapy under the supervision of ANSM, in the most relevant indications (and not exclusively in the field of bone and joint infection). Recently, the PHAG-ONE project, which is part of the PHAGE*in*LYON program, has aimed to develop, produce and use therapeutic phages targeting antibiotic-resistant *S. aureus*, *S. epidermidis* and *E. coli* to treat infections due to these bacteria. This project has been funded by French authorities, and started in September 2021.

In Europe, a new study group of the European Society of Clinical Microbiology and Infectious Diseases (ESCMID) dedicated to non-traditional antibacterial therapy, called the ESCMID Study Group for Non-Traditional Antibacterial Therapy (ESGNTA), has been recently created. This group has several objectives, and aim especially to: (i) foster the establishment of microbiological guidelines and data; (ii) support clinical use of phages either via clinical trials or compassionate use; (iii) promote the creation of academic phage therapy centers in European countries; (iv) discuss the status of phage therapy with EMA; and (v) support the knowledge in the phage lysin and other phage-related proteins research. Of note, phage-derived enzymes such as lysins, in contrast to phage therapy, have clearly to follow the classical process of medicine products.

Taken altogether, phage therapy and phage-derived proteins have huge potential to treat biofilm-associated bacterial diseases such as bone and joint infections, but not exclusively, and this is of crucial importance in the worldwide spread of antimicrobial resistance.

## Data Availability

Not applicable.
